# Host Responses in the Link Between Periodontitis and Rheumatoid Arthritis

**DOI:** 10.1007/s40496-014-0039-2

**Published:** 2014-12-24

**Authors:** Tetsuo Kobayashi, Hiromasa Yoshie

**Affiliations:** 1 General Dentistry and Clinical Education Unit, Niigata University Medical and Dental Hospital, 2-5274 Gakkocho-dori, Chuo-ku, Niigata 951-8514 Japan; 2 Division of Periodontology, Department of Oral Biological Science, Niigata University Graduate School of Medical and Dental Sciences, 2-5274 Gakkocho-dori, Chuo-ku, Niigata 951-8514 Japan

**Keywords:** Periodontitis, Rheumatoid arthritis, Cytokine, Tumor necrosis factor-alpha, Interluekin-6, Targeted therapy

## Abstract

Periodontitis and rheumatoid arthritis (RA) are common chronic inflammatory conditions and share many clinical and pathologic features. There is evidence to suggest that similar profiles of cytokine genotypes and their coding proteins are involved in the pathogenesis of periodontitis and RA. In particular, constitutive overproduction of pro-inflammatory cytokines, including tumor necrosis factor-alpha (TNF-α) and interleukin-6 (IL-6), has been implicated to play a pathologic role in the two inflammatory diseases. Results from studies with animal and human subjects have suggested an improvement of periodontal inflammatory condition after treatment with TNF-α inhibitors. Likewise, IL-6 receptor inhibition therapy has been suggested to have an effect on control of periodontal inflammation in patients with RA. In the present review, we provide an overview of studies showing the pathological role of cytokines in the linkage between periodontitis and RA, and further summarize the current studies assessing the effect of cytokine targeted therapy on periodontal condition.

## Introduction

Rheumatoid arthritis (RA) is a systemic autoimmune disease characterized by chronic inflammation and joint tissue destruction, leading to functional disability. The prevalence of RA is estimated to be approximately 0.5 % in the world [1]. Periodontitis represents a chronic inflammatory disease affecting tooth-supporting tissues, and is initiated by oral anaerobic bacteria. Similarities in the clinical and pathological features have been suggested between periodontitis and RA [2–6]. It has been reported that patients with RA are more likely to have periodontitis [3, 7–10], whereas patients with moderate-to-severe periodontitis have a higher prevalence of RA than those without periodontitis [10–12]. These bidirectional relationships between the two diseases might be related to the common host immune response as well as similar pathobiology.

Despite the differences in initiating etiological mechanisms, several polymorphisms of genes encoding cytokines have been proposed to affect connective tissue and bone metabolism in both diseases [4]. In addition, there is accumulating evidence to support the notion that both periodontitis and RA manifest as a result of persistent high levels of pro-inflammatory cytokines and their related molecules [5]. In particular, constitutive overproduction of tumor necrosis factor-alpha (TNF-α) and interleukin-6 (IL-6) has been suggested as one of the most common confounding factors for the two inflammatory diseases [6]. Moreover, novel therapeutic strategies based on pro-inflammatory cytokine blockade have been developed for the treatment of RA. Several studies with animals and the patients have evaluated the efficacy of cytokine-targeted therapy on periodontal inflammation and destruction levels.

The present review focuses on the evidence regarding the pathologic role of specific cytokine genotypes and their coding proteins in the pathogenesis of periodontitis and RA. Furthermore, we summarize the studies assessing the effect of cytokine targeted therapy on periodontal inflammatory condition in patients with RA (Table 1).

**Table 1:** Studies assessing the effect of cytokine-targeted therapy on periodontal condition in patients with rheumatoid arthritis (RA)

Authors	Targets (Biologics)	Subjects (Number)	Parameters	Summary
Pers et al. [80]	TNF (IFX)	2 groups: RA with IFX (20) and without IFX (20). Of these, RA with IFX (9) evaluated before and after 6 weeks.	PI, MGI, PBI, PD, AL	IFX increased MGI and PBI, decreased AL, and did not affect PD.
Ortiz et al. [81]	TNF and TNFR (IFX, ETN, ADA)	4 groups: TNFI+Sc/Rp (10), TNFI (10), Sc/Rp (10), and non-TNFI non-Sc/Rp (10), evaluated before and after 6 weeks.	PI, GI, BOP, PD, CAL, serum TNF-α	TNF inhibitors decreased GI, BOP, PD, and CAL, which is only shown with periodontal therapy.
Mayer et al. [82]	TNF (IFX)	3 groups: RA with IFX (10) and without IFX (10), and healthy control (10).	PI, GI, BOP, PD, Clinical AL, GCF TNF-α	IFX decreased GI, BOP, clinical AL, and GCF TNF-α levels, and did not affect PD
Mayer et al. [83]	TNF (IFX)	5 groups: RA with IFX (10), RA without IFX (12), PA (12), SSc (12,) and healthy control (10).	PI, GI, FMBS, PD, GCF TNF-α	IFX decreased GI, FMBS, and GCF TNF-α levels.
Üstün et al. [84]	TNF (IFX, ADA)	2 groups: RA with IFX (9) and RA with ADA (7), evaluated before and after 30 days.	PI, GI, BOP, PPD, CAL, saliva/GCF IL-1βIL-8, MCP-1	TNF blockers decreased GI, %BOP, GCF levels of IL-1β and IL-8, and saliva levels of IL-8 and MCP-1.
Kobayashi et al. [85]	TNF (ADA)	1 group: RA with ADA (20) evaluated before and after 3 months.	PI, GI, BOP, PD, CAL, serum TNF-αIL-6, MMP-3	ADA decreased GI, %BOP, PD, and serum levels of TNF-α, IL-6, and MMP-3, which is related to decrease in acute-phase proteins.
Kobayashi et al. [86••]	IL-6R (TCZ)	2 groups: RA with TCZ (28) and without TCZ (27), evaluated during 8 weeks.	PI, GI, BOP, PD, CAL, serum TNF-αIL-6, MMP-3	TCZ showed a greater decrease in GI, %BOP, PD, CAL, and serum levels of IL-6 and MMP-3.

*TNF* Tumor necrosis factor, *IFX* Infliximab, *TNFR* TNF receptor, *ETN* Etanercept, *ADA* Adalimumab, *IL-6R* Interleukin-6 receptor, *TCZ* Tocilizumab, *TNFI* TNF inhibitor,

*Sc/Rp* Scaling and root planing, *PA* Psoriatic arthritis, *SSc* Systemic sclerosis, *PI* Plaque index, *MGI* Modified gingival index, *PBI* Papillary bleeding index, *PD* Probing depth,

*AL* Attachment loss, *GI* Gingival index, *BOP* Bleeding on probing, *GCF* Gingival crevicular fluid, *CAL* Clinical attachment level, *FMBS* Full-mouth bleeding score,

*PPD* Probing pocket depth, *IL-1β* Interleukin-1β, *MCP-1* Monocyte chemoattractant protein-1, *MMP-3* Matrix metalloproteinase-3

## Common Genetic Components

### Case-Control Study

Susceptibility to periodontitis is influenced by genetic and environmental factors, as well as periodontopathic bacteria [13]. The evidence for genetic susceptibility to periodontitis comes from twin studies showing that half of the population variance in periodontitis is attributable to genetic factors [14, 15]. It is also established that genetic and environmental factors participate in the pathogenesis of RA [16]. A family association study indicated that the standardized incidence ratio for RA in offspring of affected parents was 3.02 [17]. The genetic contribution to RA in the population accounted for approximately 60 % of the variation in liability to disease in a twin study [18]. These findings suggest that genetically determined variances in host immune responses are important determinants for susceptibility for both periodontitis and RA.

The first approach to find common genetic risk factors was conducted in a case-control study with Danish white adults, documenting that frequencies of IL-1A -889 and +4845, and IL-1B -511 and +3954 genotypes were similar among localized and generalized aggressive periodontitis (LAgP and GAgP), juvenile idiopathic arthritis, RA, and healthy control groups [19]. However, the differences in cytokine levels between the IL-1 genotypes were observed within all four disease groups, but not within the control group [19]. These findings suggest a shared genetic background of IL-1 for cytokine profiles of patients with AgP and RA. Another case-control study with Japanese adults demonstrated that the distributions of IL-1B +3954 genotypes and haplotypes of IL-1A +4845 and IL-1B +3954 were unique to patients with RA and periodontitis compared with those with periodontitis and healthy individuals [20]. These observations are supported by the results of another study evaluating 16 cytokine gene polymorphisms encoding IL-1, IL-2, IL-4, IL-6, IL-10, TNF-α, and transforming growth factor-β 1 [21].

Pathologic expression of genes in individuals with periodontitis and RA might be reflected in peripheral blood mononuclear cells (PBMCs), because of their close relation to the pathogenesis of inflammatory diseases. Microarray analyses have revealed that 53 differentially expressed candidate genes were identified in PBMCs from individuals with LAgP. Of these, 14 genes were associated with cytokine responses including IL-1B and IL-6 [22], which is consistent with the results of the above-mentioned genetic studies [20, 21]. The validation of these genetic data using real-time reverse transcription-polymerase chain reaction (RT-PCR) confirmed that Toll-like receptor 2 (TLR2) gene exhibited a higher expression by PBMCs from individuals with LAgP and those with RA compared with healthy controls [24]. These findings are supported by the results of other studies [23, 24] demonstrating an increased expression of TLR2 in periodontitis-affected gingival tissue as well as blood monocytes and synovial tissue macrophages from patients with RA. It is, therefore, suggested that elevated gene expressions for IL-1B and TLR2 may constitute a common risk factor for periodontitis and RA. However, there has been some difficulty identifying the disease-specific genes, because no direct and simple correlation has been obtained between PBMCs transcript and protein levels of IL-1A, -1B, -1 receptor antagonist (IL-1RN), IL-6, IL-10, TNFA, and TNF receptor I and II, determined by RT-PCR and sensitive enzyme-linked immunosorbent assay (ELISA) [25].

Epigenetic variability is a crucial mechanism in regulation of the production of pro-inflammatory cytokines relevant to the pathogenesis of inflammatory diseases such as RA and periodontitis　[26]. Two major epigenetic mechanisms of interest are the post-transcriptional modification of histone proteins in chromatin and methylation of DNA which are regulated by distinct, but coupled, pathways. Analysis of bisulfite genomic sequences has revealed that a total of 19 CpG motifs were identified from -1200 bp to +27 bp in IL-6 promoter region, and that the methylation levels of the CpG motif at -74 bp were lower in patients with chronic periodontitis (CP) and RA than in healthy controls [27]. It has also been demonstrated that levels of serum IL-6 and IL-6 production by PBMCs were different between individuals with and without the methylation at -74 bp [27]. These results suggest that the hypomethylated status of a single CpG in the IL-6 promoter region leads to increased levels of serum IL-6, implicating a role in the pathogenesis of CP and RA.

Gene association studies are of limited value, despite their effectiveness in detecting genes underlying common and more complex diseases such as periodontitis where the genetic risk is relatively small [28, 29]. The first major concern related to many of the gene association studies carried out to date is that relatively small size of the participants, which results in a large potential false-positive and false-negative results, and exhibit a lack of statistical power to properly detect association. Another important issue is the difficulty in defining the disease phenotypes. It is difficult to distinguish AgP from CP due to their similarities in the clinical symptoms, which makes interpretation and replication of the case-control studies difficult. A third concern is that the study participants should be carefully matched to ethno-geographic origin, because the frequencies of genotype and allele may have ethnic heterogeneity. For example, frequencies of the rare allele in the IL-1A +4845, IL-1B +3954, and IL-1 variable number of tandem repeats genes are extremely low in Asian in contrast to white populations [28]. Furthermore, it should be emphasized that such association studies only assess the candidate risk genes selected according to the hypotheses based on the molecular biological mechanisms, but may overlook other genes involved in the host responses. Collectively, it would be more effective to apply a hypothesis-free approach, genome-wide association study (GWAS) to enable the parallel screening of common risk genes in relation to periodontitis and RA [30•, 31, 32].

### Genome-Wide Association Study (GWAS)

Despite performing many different GWAS for almost all human diseases, only three GWAS have been reported in relation to periodontitis [33–35]. In 2010, the first GWAS demonstrated an association of the glycosyltransferase 6 domain containing one gene on chromosome 9q34.3 with AgP, which was repeatedly replicated through a clinical network across Germany (283 AgP and 972 control) and the Netherlands (164 AgP and 368 control) [33]. It has also been demonstrated that the rare allele of SNP rs 1537415 resulted in an impaired binding of the transcription factor, GATA binding protein 3 (GATA-3), which is related to the transcriptional control of T-helper 2 cell differentiation. These results suggest that this locus constitutes an important susceptibility factor, and that GATA-3 is a potential signaling component in the pathophysiology of AgP. The second GWAS conducted in 2,681 patients with CP and 1,823 control individuals of Americans of European ancestry did not identify any specific gene and locus in relation to CP [34], which is consistent with the results of the third GWAS assessing 4,032 German individuals with mixed periodontal condition, ranging from no or mild CP to moderate and severe CP [35].

On the other hand, two large meta-analyses of GWAS have been performed for identification of RA risk loci independently in different populations [31, 32]. The first large meta-analysis of GWAS was conducted in 5,539 autoantibody-positive individuals with RA and 20,169 controls of European descent, followed by replication of in an independent set of 6,768 RA cases and 8,806 controls [31]. In these analyses, seven new RA risk alleles, i.e. *IL6ST*, *SPRED2*, *RBPJ*, *CCR6*, *IRF5*, and *PXK*, were identified at genome-wide significance, and three associated RA risk loci, i.e. *IL2RA*, *CCL21*, and *AFF3*, were refined [31]. The second large meta-analysis of GWAS was conducted in a Japanese population including 4,074 individuals with RA and 16,891 controls, followed by a replication in 5,277 RA cases and 21,684 controls [32]. These analyses identified nine loci newly associated with RA, which includes *B3GNT2*, *ANXA3*, *CSF2*, *CD83*, *NFKBIE*, *ARID5B*, *PDE2A-ARAP1*, *PLD4*, and *PTPN2* [32]. In addition, a multi-ancestry comparative analysis of 46 risk loci conducted between the above-mentioned Japanese and European descents showed a significant association with RA at 22 loci in Japanese and at 36 loci in Europeans, with 14 loci being shared between the populations [32].

Collectively, these observations suggest difficulties in identifying common genetic risk factors for periodontitis and RA, despite a large number of genetic case-control studies and GWAS in individuals with both diseases. In the future, it will be necessary to identify causal genetic variants and their functional characterization, which might be achieved by use of new technologies and innovative research strategies.

## Common Pro-inflammatory Cytokines

Periodontitis and RA have been suggested to exhibit a common pathophysiology characterized by persistent high levels of pro-inflammatory cytokines and reduced levels of anti-inflammatory mediators in the compartment adjacent to bone [5]. In particular, it has been suggested that the excess production and release of pro-inflammatory cytokines including TNF-α and IL-6 are involved in the soft and hard tissue destruction in both inflammatory diseases [36–39].

### TNF-α and Related Receptors

TNF-α plays a critical role in the host response against a wide range of bacterial infections, which contributes to both innate and adaptive immune response [40, 41]. The majority of antimicrobial and inflammatory effects of TNF-α are mediated through the TNF-α receptor p55, whereas TNF-α signaling through the p75 receptor acts to attenuate the inflammatory response [42]. A number of animal studies have indicated a significant role for TNF-α in the alveolar bone loss that is a main characteristic of periodontitis [36, 37, 43–45]. It has been reported that *Porphyromonas gingivalis*-induced osteoclastogenesis is reduced in mice deficient in TNF-α receptor p55 and p75 compared to wild-type mice [43]. This suggests that osteoclast formation resulted from stimulation of the host response rather than from the direct effect of bacterial products. In particular, TNF-α receptor p55-knockout mice develop a less severe periodontitis in response to oral infection with *Aggregatibacter actinomycetemcomitans* (previously *Actinobacillus actinomycetemcomitans*), characterized by less alveolar bone loss and inflammatory reaction compared with wild-type controls [44]. Furthermore, subcutaneous administration of recombinant human TNF-α accelerates the progression of experimental periodontitis [45].

With regard to human periodontitis, an immunohistochemistry study showed increased levels of TNF-α receptor p55 and p75 expression and TNF-α positive cells in the gingival tissue from patients with adult periodontitis compared with the healthy controls [46]. In addition, the concentration of TNF-α in gingival crevicular fluid (GCF) from patients with CP and AgP were higher than those in GCF from healthy control individuals [47]. Furthermore, the total amounts of TNF-α and soluble TNF receptor 1 and 2 in GCF elevated with increasing probing depth (PD) in both site-based and subject-based analyses [48]. These results obtained from both animal and human studies demonstrate clearly that TNF-α plays a central role in the host inflammatory reaction, which is related to the breakdown of alveolar bone as well as loss of connective tissue attachment.

The significant role of TNF-α in the pathogenesis of RA has been well documented [38, 39]. Relevance of TNF-α to periodontal inflammatory condition has also been implicated in patients with RA [49–51]. TNF-α levels in unstimulated whole blood cell culture were higher in patients with RA than those in healthy controls [49]. Serum levels of TNF-α were increased in patients with RA than those in age-, gender-, smoking status-, and periodontal condition-balanced control individuals [50]. Interestingly, it was shown that serum levels of TNF-α was positively correlated with RA activity and bleeding on probing (BOP) in patients with RA [50]. Moreover, RA patients with high plasma levels of time-averaged TNF-α had a higher frequency of BOP as well as increased PD and clinical attachment level (CAL) compared with those with low plasma levels of TNF-α [51]. These observations suggest that periodontal inflammation may be related to high levels of systemic and local TNF-α in patients with RA.

### IL-6 and Signaling Mediators

IL-6 is a multifunctional pro-inflammatory cytokine that has a wide range of biological activities in various target cells and regulates immune responses, acute phase reactions, hematopoiesis, and bone metabolism [52, 53]. IL-6 signaling is mediated through two functional membrane proteins: an 80 kDa ligand-binding chain (IL-6 receptor [IL-6R], IL-6R α-chain, or CD126) [54] and a 130 kDa non-ligand-binding signal-transducing chain (glycoprotein 130 [gp130], IL-6R β-chain, or CD130) [55]. In cells with sufficient membrane-bound IL-6R, IL-6 binds to these receptors, followed by the IL-6/IL-6R complex induced-homodimerization of the gp130 molecule, and forms a high-affinity functional receptor complex IL-6/IL-6R/gp130 [56]. However, under insufficient cell-surface IL-6R, IL-6 signal transduction starts with the binding of IL-6 to the free soluble IL-6R (sIL-6R), which lacks the membrane and intracytoplasmic portion of the membrane-bound IL-6R molecule [55, 56].

Several studies have reported the systemic and local production of IL-6 and its signaling mediators in patients with periodontitis [57, 58•]. High levels of IL-6-specific mRNA have been observed in gingival mononucliar cells (GMCs) from patients with adult periodontitis [59]. IL-6 protein is also produced in the culture supernatants of GMCs, but not in those of PBMCs in the same patients [59]. Furthermore, the expressions of mRNA for IL-6, IL-6R, and gp130, as well as those of proteins for IL-6 and sIL-6R can be detected in inflamed gingival tissue [60, 61]. In accordance with these findings, expressions of IL-6-specific mRNA and protein can be detected in human periodontal ligament fibroblasts and gingival fibroblasts upon stimulation with lipopolysaccharides from periodontopathic bacteria such as *P. gingivalis* and *A. actinomycetemcomitans* [62–64]. It has also been documented that systemic (serum) and local (GCF and saliva) levels of IL-6 are increased in patients with periodontitis than those in the healthy controls [65–68]. Serum levels of IL-6 appear to be reduced after intensive periodontal treatment including oral hygiene instruction, subgingival scaling, and root planing [68–70].

The pathological role of IL-6 has been documented in patients with RA as well [38, 39]. It has been documented that a trend toward increase in GCF levels of IL-6 was observed in patients with RA [71]. Increased levels of IL-6 were also observed in unstimulated whole blood cell culture of patients with RA compared with those of the healthy controls [49]. Another study indicated that serum levels of IL-6 were higher in patients with RA than those in age-, gender-, smoking status-, and periodontal condition-balanced healthy control individuals, and also showed that serum levels of IL-6 were positively correlated with RA activity [50]. These observations suggest that systemic and local produced IL-6 may play a role in regulating periodontal inflammation in patients with RA.

## Cytokine-Targeted Therapies

It has been well recognized that both periodontitis and RA are associated with persistent high levels of pro-inflammatory cytokines including TNF-α, IL-1, and IL-6 [4, 5, 36–39]. This leads to the hypothesis that pro-inflammatory cytokine-targeted pharmacologic treatment may reduce systemic and local levels of these cytokines, and concomitantly improve periodontal inflammatory condition. Novel therapeutic strategies based on TNF-α and IL-6 blockade have been developed, and are currently used for the treatment of RA [72•, 73]. To date, there have been several studies evaluating in vitro and in vivo efficacy of TNF-α- and IL-6R-targeted therapies in the treatment of periodontitis.

Animal Studies

Significant inhibitory effects of IL-1 and TNF antagonists on periodontal inflammatory and destructive responses have been demonstrated in a *Macaca fascicularis* primate model of experimental periodontitis [74]. The results indicated that injection of soluble receptors to IL-1 and TNF inhibited by approximately 80 % the recruitment of inflammatory cells in close proximity to bone, and also reduced the formation of osteoclasts by 67 % and the amount of bone loss by 60 %, respectively [74]. Likewise, blockade of IL-1 and TNF resulted in an inhibitory effects on the progression of inflammatory cell infiltration toward alveolar bone in the same animal model [75]. In addition, the histomorphometric analysis indicated that IL-1 and TNF antagonists reduced the loss of connective tissue attachment by approximately 51 % and the loss of alveolar bone height by almost 91 % in the same experimental periodonttiis model [76]. However, the effects of local injection of IL-1 and TNF soluble receptors proved different between the early and late phases of periodontal wound healing in a non-human primate model [77]. Short-term blockade of IL-1 and TNF may facilitate periodontal wound healing, whereas the prolonged blockade may have adverse effects [77]. Furthermore, recent studies showed that anti-TNF-α antibody treatment improved the host response to *Porphyromonas gingivalis* and was related to reduced serum levels of TNF-α and IL-6 in mice [78, 79].

Human Trials

The effect of cytokine-targeted therapy on periodontal condition in patients with RA has been reported in several studies [80–85, 86••]. A summary of study methods and results obtained from each study are presented in a chronological order (Table 1). First, Pers et al. evaluated periodontal condition in 20 French patients with RA who had received infliximab (IFX, a chimeric mouse/human anti-TNF-α monoclonal antibody) every 6 weeks for more than 22 months and 20 race-, age-, gender-, and smoking status-balanced patients with RA who had not received IFX medication [80]. Of 20 patients in the control group, nine were subjected to assessment before and after IFX therapy. The results showed that IFX increased modified gingival and papillary bleeding indices and decreased attachment loss, but did not affect PD [80]. The second comparative clinical study conducted by Oritz et al. reported that medication of TNF inhibitors including IFX, etanercept (ETN, a recombinant fusion protein linked to human type II TNF receptor-Fc portion), or adalimumab (ADA, a fully humanized anti-TNF-α monoclonal antibody) resulted in an improvement of gingival index (GI), BOP, PD, and CAL, which is only shown with periodontal therapy [81]. Mayer et al. indicated that patients with RA who received IFX had lower periodontal indices and GCF TNF-α levels [82, 83]. In addition, Üstün et al. showed that TNF inhibitors such as IFX and ADA decreased periodontal inflammatory indices, and also lowered GCF levels of IL-1 β and IL-8, and saliva levels of IL-8 and monocyte chemoattractant protein-1 [84]. Recently, Kobayashi et al. demonstrated a beneficial effect of ADA therapy on periodontal inflammatory condition in patients with RA [85]. It was proposed that this might be related to decrease in serum levels of CRP, matrix metalloproteinase-3 (MMP-3), IL-6, TNF-α, and acute phase proteins [85]. On the other hand, there has been only one study assessing IL-6R inhibition therapy with tocilizumab (TCZ, a humanized monoclonal anti-human IL-6R antibody with specificity for the soluble and membrane-expressed IL-6R) on periodontal condition in patients with RA and CP [86••]. The results showed that the patients with RA who received TCZ exhibited a greater decrease in GI, BOP, PD, CAL, and serum levels of IL-6 and MMP-3 than the control patients [86••].

## Conclusions

It has been recognized for some time that periodontitis and RA share many common pathologic features, which may be based on the similarities in host response. In particular, constitutive overproduction of pro-inflammatory cytokine, TNF-α, and IL-6 has been implicated as one of the most common confounding factors for the two inflammatory diseases. Results from animal and clinical intervention studies have suggested an improvement of periodontal inflammatory condition after treatment with TNF-α and IL-6 receptor inhibitors. However, the clinical studies carried out to date have had very low numbers of subjects and short time for observation. Therefore, more extensive studies in a larger patient group would be necessary to better assess the therapeutic efficacy of pro-inflammatory cytokine blockade on periodontitis.
